# Lysine Distinctively Manipulates Myogenic Regulatory Factors and Wnt/Ca^2+^ Pathway in Slow and Fast Muscles, and Their Satellite Cells of Postnatal Piglets

**DOI:** 10.3390/cells13070650

**Published:** 2024-04-08

**Authors:** Xiaofan Wang, Xiaoyin Zong, Mao Ye, Chenglong Jin, Tao Xu, Jinzeng Yang, Chunqi Gao, Xiuqi Wang, Huichao Yan

**Affiliations:** 1 College of Animal Science, South China Agricultural University, State Key Laboratory of Swine and Poultry Breeding Industry, National Engineering Research Center for Breeding Swine Industry, Guangdong Laboratory for Lingnan Modern Agriculture, Guangdong Provincial Key Laboratory of Animal Nutrition Control, Guangzhou 510642, China; xxw033@scau.edu.cn (X.W.); zongxiaoyin@stu.scau.edu.cn (X.Z.); ym-yf@trsgroup.cn (M.Y.); jincl@scau.edu.cn (C.J.); xutaott@outlook.com (T.X.); cqgao@scau.edu.cn (C.G.); xqwang@scau.edu.cn (X.W.); 2 Institute of Animal Science, Guangdong Academy of Agricultural Sciences, Key Laboratory of Animal Nutrition and Feed Science (South China) of Ministry of Agriculture, State Key Laboratory of Swine and Poultry Breeding Industry, Guangdong Public Laboratory of Animal Breeding and Nutrition, Guangdong Key Laboratory of Animal Breeding and Nutrition, Guangzhou 510640, China; 3 Department of Human Nutrition, Food and Animal Sciences, University of Hawaii at Manoa, Honolulu, HI 96822, USA; jinzeng@hawaii.edu

**Keywords:** myogenic differentiation, satellite cell, Wnt/Ca^2+^ pathway, lysine

## Abstract

Muscle regeneration, representing an essential homeostatic process, relies mainly on the myogenic progress of resident satellite cells, and it is modulated by multiple physical and nutritional factors. Here, we investigated how myogenic differentiation-related factors and pathways respond to the first limiting amino acid lysine (Lys) in the fast and slow muscles, and their satellite cells (SCs), of swine. Thirty 28-day-old weaned piglets with similar body weights were subjected to three diet regimens: control group (d 0–28: 1.31% Lys, n = 12), Lys-deficient group (d 0–28: 0.83% Lys, n = 12), and Lys rescue group (d 0–14: 0.83% Lys; d 15–28: 1.31% Lys, n = 6). Pigs on d 15 and 29 were selectively slaughtered for muscular parameters evaluation. Satellite cells isolated from fast (*semimembranosus*) and slow (*semitendinosus*) muscles were also selected to investigate differentiation ability variations. We found Lys deficiency significantly hindered muscle development in both fast and slow muscles via the distinct manipulation of myogenic regulatory factors and the Wnt/Ca^2+^ pathway. In the SC model, Lys deficiency suppressed the Wnt/Ca^2+^ pathways and myosin heavy chain, myogenin, and myogenic regulatory factor 4 in slow muscle SCs but stimulated them in fast muscle SCs. When sufficient Lys was attained, the fast muscle-derived SCs Wnt/Ca^2+^ pathway (protein kinase C, calcineurin, calcium/calmodulin-dependent protein kinase II, and nuclear factor of activated T cells 1) was repressed, while the Wnt/Ca^2+^ pathway of its counterpart was stimulated to further the myogenic differentiation. Lys potentially manipulates the differentiation of porcine slow and fast muscle myofibers via the Wnt/Ca^2+^ pathway in opposite trends.

## 1. Introduction

Skeletal muscle is essential for physical movement and performs extensive physiological functions such as glucose uptake, protein reservoir, and metabolic activity to sustain body health [1,2]. Muscle regeneration, representing a homeostatic process, relies mainly on the myogenic progress of resident satellite cells (SCs), modulated by multiple physical and nutritional factors. 

Muscle types, including fast (e.g., *semimembranosus*) and slow (e.g., *semitendinosus*) muscles, varying in metabolic and physiological features, drive SC differentiation differently [3,4,5]. Khodabukus et al. isolated and cultured SCs from soleus (slow) and tibialis anterior (fast) muscles and regained the myofibers resembling the contractile and metabolic properties of the original muscle source [6]. Notably, SCs are twice as abundant in slow muscles as in their fast counterparts but possess subtle contributions to the different myofiber formations [7]. Instead, the main modulating factors of myofiber differentiation are myogenic regulatory factors (MRFs) comprising of myogenin and myogenic regulatory factor 4 (MRF4) [8]. MRFs have differential expression properties in fast and slow muscles [9,10,11]. They are expressed in a temporally continuous process to mediate developmental and regenerative myogenesis. Myogenin and MRF4 expressions mark the beginning of terminal differentiation [8], and notably, over- or underexpressions of myogenin repress skeletal muscle development. How MRFs modulate SCs’ progress in fast and slow muscles is yet to be determined. 

Moreover, extensive studies have demonstrated the critical roles of both canonical Wnt/β-catenin and noncanonical Wnt/planar cell polarity (PCP) pathways in SC proliferation and symmetric amplification [12,13]. In 2018, Zhang et al. illuminated the indispensable role of Wnt/Ca^2+^ pathways to SC differentiation, which was recapitulated by the phenomenon that the expression of Wnt5a, the main Wnt/Ca^2+^ ligand, increases as SC differentiation continues [14]. However, the cellular Ca^2+^ concentration differing in slow and fast muscle cells potentially drives a different dependency on the Wnt/Ca^2+^ pathway and SC proliferation, and its underlying mechanism in different muscle types was overlooked before. 

Dystrophy can cause severe myasthenia and hypotonia. Lysine (Lys), the first essential amino acid in corn–soybean-based diets for swine, limits the production of endogenous carnitine and further affects muscular development and growth [15]. In addition, previous investigations highlighted the critical role of Lys in modulating the amplification and migration of SCs as a signaling factor [16,17,18]. However, the mechanisms through which Lys modulates muscle myogenic differentiation in different types of skeletal muscles remain elusive. This led us to investigate, for the first time, the Lys-Wnt/Ca^2+^ axis in SC differentiation in both slow and fast muscles.

## 2. Materials and Methods

### 2.1. Ethics Statement

All animals were managed according to the guidelines for the Care and Use of Laboratory Animals of South China Agricultural University (Guangzhou, China). The experiments were approved by the Animal Ethics Committee of South China Agricultural University (Guangzhou, China) with AECSCAU: SCAU0158.

### 2.2. Animal Management and Study Design

Thirty 28-day-old weaned piglets (Duroc × Landrace × Large White) with similar body weights (BW; 8.42 ± 0.07 kg) were chosen for the 28-day experiment. The swine farm was thoroughly cleaned and disinfected before the study. Hygiene management and the vaccination strategy followed the standard facility maintenance procedures. Piglets were allowed to acclimate for five days to the new facility and diet before the onset of the study. Feed and water were provided ad libitum throughout the study. The ingredient composition and nutritional levels of the diets are illustrated in Table S1.

On day 0, the piglets were randomly assigned into the control (1.31% Lys, n = 12) and Lys-deficient groups (0.83% Lys, n = 18). On d 15, six piglets closest to the BW average from each group were selected for sacrifice. Six piglets in the Lys-deficient group were randomly selected and transferred to the control diet, while the remaining six pigs were maintained on a Lys-deficient diet until study completion. On d 29, all piglets were slaughtered for sample and data collections. 

### 2.3. Muscle Sample Collections and Processing

On the day of animal sacrifice, pigs were sacrificed via anterior vena cava bleeding. Fore- and hindlimbs were cleaned with alcohol wipes. Then, all *semimembranosus* (fast) and *semitendinosus* (slow) muscles were completely dissected for mass assessment. Meanwhile, muscle specimens from each limb were also collected and stored at −80 °C for mechanism investigations. Strips of fresh muscle samples were excised along the muscle fibers followed by soaking in high-sucrose solution for rapid dehydration for 24 h at room temperature. The specimens were then immersed in pre-cooled isopentane for 30 min and frozen at −80 °C for biopsy slices preparation. 

### 2.4. Protein Extraction and Assay

For each specimen, a total of 50 g of muscle together with 600 μL of lysis buffer (BioTeke, Beijing, China), 400 μL of pure water, and 10 μL of the protease inhibitor phenylmethanesulfonyl fluoride (PMSF; Sigma-Aldrich, St. Louis, MO, USA) were subjected to thorough homogenization (3 times × 60 Hz for 1 min). Specimens were then maintained on ice for 30 min to obtain the cell lysates and then centrifuged twice at 12,000 rpm for 10 min to collect the supernatant. Protein levels were determined following the instruction of the Pierce BCA Protein Assay Kit (Thermo Fisher, Rockford, IL, USA). All protein samples were manipulated to the lowest concentration before downstream evaluation, which was further validated by sodium dodecyl-sulfate polyacrylamide gel electrophoresis (SDS-PAGE Gel Quick Preparation Kit, Beyotime, Shanghai, China). 

### 2.5. Western Blotting

The protein mixture (20% in premixed loading buffer) was denatured by boiling for 10 min, and then the proteins were separated through 10% SDS-PAGE. The separated proteins were blotted onto polyvinylidene fluoride membranes (PVDF; Millipore, Burlington, MA, USA). The membranes were sequentially incubated with specific primary (targeting the protein of interest) and secondary antibodies (specific to the host species of the primary antibody; Table S2). Immunoreactivity was visualized using the Beyo ECL Plus chemiluminescence detection kit (Millipore, Burlington, MA, USA) and the FluorChem M system (Protein Simple, San Jose, CA, USA). The band density was analyzed using ImageJ analysis software (version 1.8.0 112, https://imagej.nih.gov; accessed on 20 March 2022) after background noise exclusion. 

### 2.6. Immunofluorescence Staining

Muscle specimens were embedded with OTC compound and processed into 5 μm thick pieces. Then, the specimens were permeated with 0.1% Triton X-100 for 10 min, followed by PBS rinses conducted three times. After blocking with 5% bovine serum albumin for 30 min, the specimens were sequentially incubated with primary (4 °C overnight) and secondary antibodies (2 h at room temperature; 40 r/min; Table S3). The membranes were rinsed with PBS (3 × 5 min) to remove the remaining antibodies after each incubation cycle on a decoloring shaker (Kylin-Bell, Haimen, Jiangsu, China). Cellular nuclei were labeled using 1 µg/µL 4′,6-di-amidino-2-phenylindole (DAPI, Sigma-Aldrich, St. Louis, MO, USA) for 5 min at room temperature. Images were obtained using immunofluorescence microscopy. The fixation of SCs in cell culture used 4% paraformaldehyde instead. 

### 2.7. Isolation and Culture of SCs

SCs were isolated following the procedures described previously [19]. Briefly, *semimembranosus* and *semitendinosus* muscles were dissected from 3~5-day-old piglets and were processed into 1 mm^3^ pieces to release the SCs. The cell mixtures were then transferred to culture media [Dulbecco’s modified Eagle’s medium/nutrient mixture F-12 (DMEM/F-12, Thermo Fisher, Waltham, MA, USA) supplemented with 10% fetal bovine serum (FBS, Thermo Fisher, Waltham, MA, USA)] and spun at 1000 r/min for 15 min to collect the supernatant. Collagenase type II (Gibco, Grand Island, NY, USA) and neutral protease (Gibco, Grand Island, NY, USA) were supplemented to digest muscle fibers for 3 h to facilitate the SCs isolation. The purification of SCs was further achieved by red blood cells lysis and fibroblast exclusion, which was leveraged on their rapid adhesion capability. SCs were maintained in DMEM/F-12 medium supplemented with 10% FBS at 37 °C and 5% CO_2_. 

### 2.8. SCs Response to Lys Deficiency

Upon 90% confluency, SCs were transferred to Lys- and FBS-free medium. After 6 h of starvation, cells were trypsinized and split into three groups and cultured with either standard (Con: 10% FBS and 2% HS, Lys = 0.52 mmol/L) or Lys-deficient medium (Lys deficiency: 10% FBS and 2% HS, Lys = 0.02 mmol/L). At 48 h, the Lys-deficient group was replaced with Lys-sufficient medium, and incubation continued for another 48 h, noted as the Lys rescue group. Culture conditions of Con and Lys-deficient groups remained the same during 48–96 h of incubation at 37 °C and 5% CO_2_.

### 2.9. Intracellular Ca^2+^ Assay

After 48 h of incubation under Lys-deficient conditions, SCs were rinsed three times with PBS and supplemented with 2 μM Fluo-4 AM Ca^2+^ probes incubation at 37 °C and 5% CO_2_ for 30 min. The SCs were then rinsed twice and incubated with 2 μM Fluo-4 AM Ca^2+^ probes and a 2.5 mM probenecid sodium mixture for 20 min. After the solution was removed, the SCs cells were subjected to a treatment of either PBS or 62 mM Lys solution; then, the time-lapse image of fluorescence was acquired every 5 s with a Nikon Eclipse Ti-U microscope (Nikon, Melville, NY, USA). The changes in fluorescence intensity were analyzed with the built-in Nikon imaging software.

### 2.10. Statistical Analysis

Data were processed and analyzed using Student’s *t*-test and one-way ANOVA using SPSS (version 22.0) software. Pairwise comparisons among Con, Lys deficiency, and Lys supplement groups were performed using the Tukey test. The results were expressed as mean ± SEM. Differences between treatments were considered statistically significant when *p* < 0.05 (*) and extremely significant when *p* < 0.01 (**). A tendency of difference (#) was denoted when 0.05 < *p* < 0.10. 

## 3. Results

### 3.1. Dietary Lys Affect the Development of Both Fast (Semimembranosus) and Slow (Semitendinosus) Muscles

**Figure 1:**
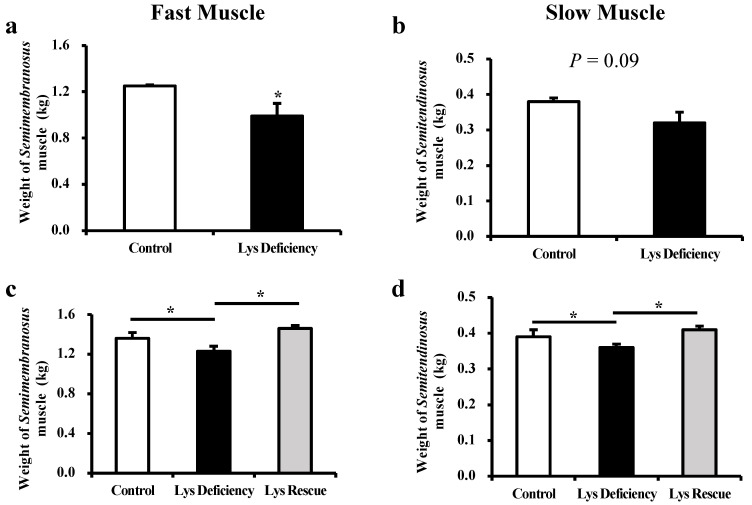
Lys deficiency inhibits fast (*semimembranosus*) and slow (*semitendinosus*) muscle growth in weaned piglets. Mass of fast (**a**) and slow muscle (**b**) in piglets weaned on day 15 of the study; mass of fast (**c**) and slow muscle (**d**) in piglets weaned on day 29 of the trial, n = 6, * indicates *p* < 0.05, same below.

The piglets had reduced total muscle mass of both fast (1.25 ± 0.01 vs. 0.99 ± 0.11 kg; *p* < 0.05; Figure 1a) and slow (0.38 ± 0.01 vs. 0.32 ± 0.03 kg; *p* = 0.09; Figure 1b) muscles after being fed the Lys-deficient diet for two weeks, and this pattern continued until the study’s completion on d 29 (*p* < 0.05; Figure 1c,d). The supplementation of sufficient Lys in the diet for two weeks (d 15–29) reversed the detrimental effect that was induced by Lys deficiency in both types of muscle (*semimembranosus* muscle: 1.23 ± 0.05 vs. 1.46 ± 0.03 kg; *p* < 0.05; *semitendinosus* muscle: 0.36 ± 0.01 vs. 0.41 ± 0.01 kg in Lys deficiency and Lys rescue groups, respectively; *p* < 0.05; Figure 1c,d), indicating the essential role of dietary Lys in guarding the muscle development of nursery piglets. 

### 3.2. Lys Deficiency and Rescue Initiates Opposite Mechanism in Fast and Slow Muscles

**Figure 2:**
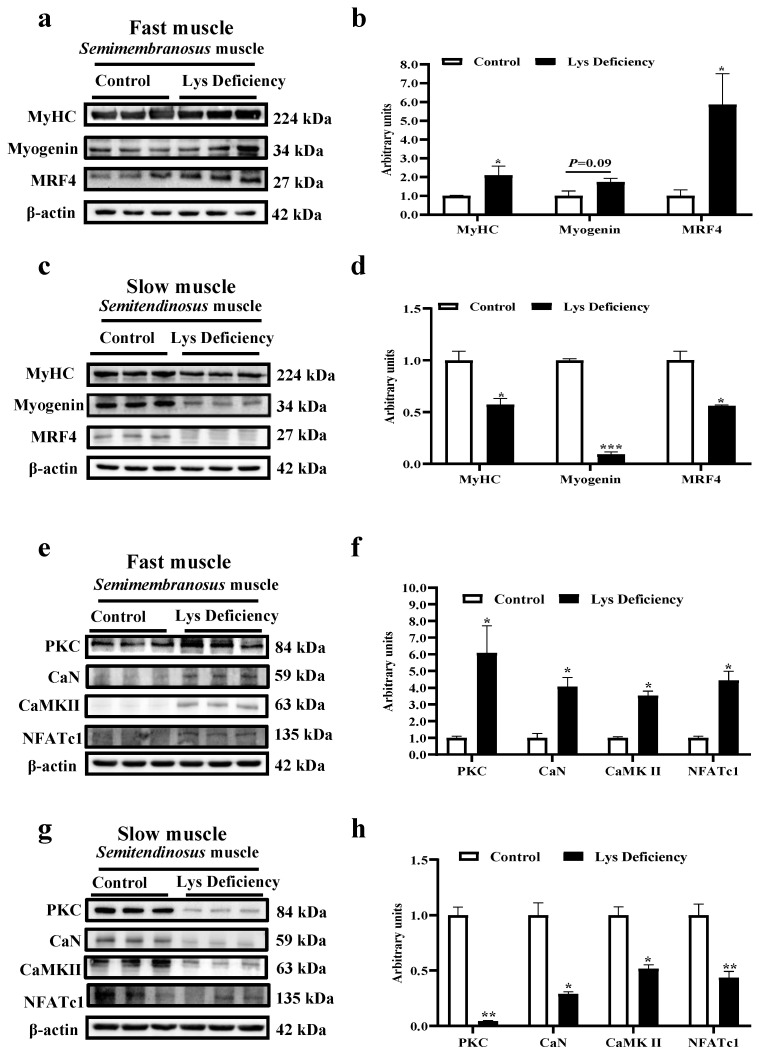
Effects of Lys deficiency on differentiation, Wnt/Ca^2+^ pathway, and muscle fiber ratio in *semimembranosus* and *semitendinosus* muscles. Expression assays of MyHC and key proteins (myogenin and MRF4) of the myogenic factor family in fast (*semimembranosus*; **a**,**b**) and slow (*semitendinosus*; **c**,**d**) muscles on d 15. Expression assays of key proteins (PKC, CaN, CaMKII, and NFATc1) of the Wnt/Ca^2+^ pathway in fast (*semimembranosus*; **e**,**f**) and slow (*semitendinosus*; **g**,**h**) muscles on d 15. Three independent trials, each with n = 3, * indicates *p* < 0.05, ** indicates *p* < 0.01, *** indicates *p* < 0.001.

On d 15, Lys deficiency increased MyHC and MRFs expressions (myogenin and MRF4; *p* < 0.05, = 0.09 and < 0.05, respectively) in fast muscles (Figure 2a,b) but significantly hindered their abundances in slow muscles (Figure 2c,d; *p* < 0.05). This muscle-type-dependent pattern was also detected in the Wnt/Ca^2+^ pathway: expressions of key proteins including protein kinase C (PKC), calcineurin (CaN), calcium/calmodulin-dependent protein kinase II (CaMKII), and nuclear factor of activated T cells 1 (NFATc1) increased on d 15 in the fast muscle of Lys-deficient pigs (Figure 2e,f; *p* < 0.05) but were significantly decreased in the slow muscle (Figure 2g,h; *p* < 0.05). 

**Figure 3:**
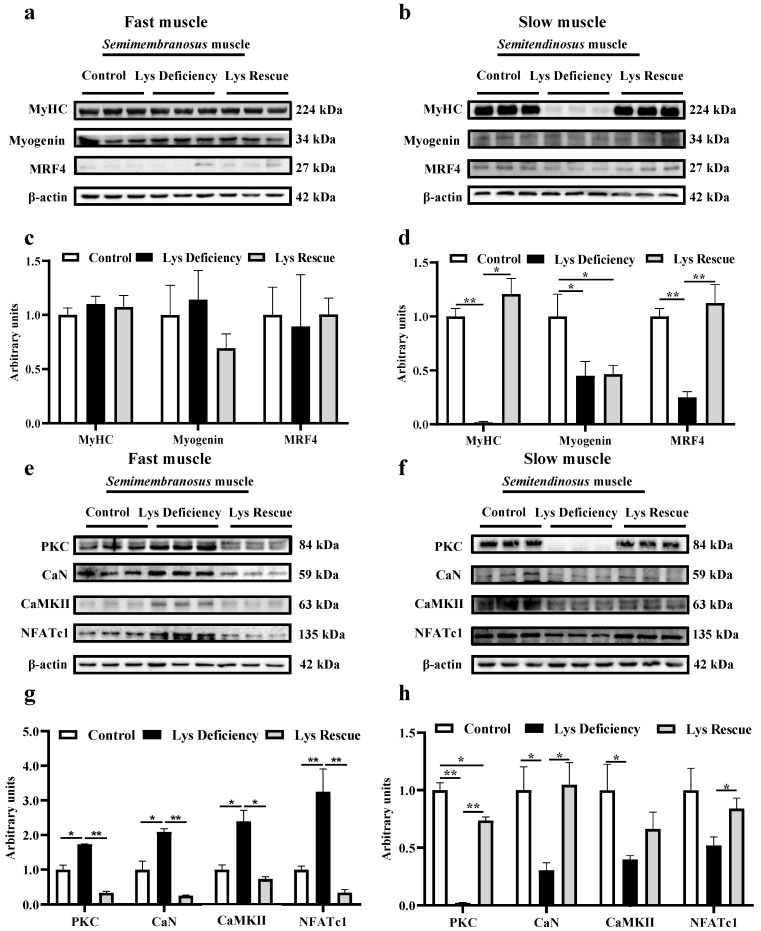
Lys re-supplementation restores the muscle differentiation and the repressed Wnt/Ca^2+^ pathway induced by Lys deficiency in slow muscle and suppresses the Wnt/Ca^2+^ pathway in fast muscle. Expression assays of MyHC and key proteins (Myogenin, and MRF4) of the myogenic factor family in fast (*semimembranosus*; **a**,**c**) and slow (*semitendinosus*; **b**,**d**) muscles on day 29 of the trial. Expression assays for key proteins of the Wnt/Ca^2+^ pathway (PKC, CaN, CaMKII, and NFATc1) in fast (*semimembranosus*; **e**,**g**) and slow (*semitendinosus*; **f**,**h**) muscles on test day 29. * indicates *p* < 0.05, ** indicates *p* < 0.01.

From d 15–29, the replacement of the diet with sufficient Lys significantly stimulated the expressions of MyHC and myofiber differentiation-modulating factors MRF4 and stimulated myogenin abundantly in *semitendinosus* muscle, but had no effect on these expressions in *semimembranosus* muscles (Figure 3a–d). Regarding the Wnt/Ca^2+^ pathway, Lys rescue remarkably hindered the pathway-associated key molecules in the fast muscle (Figure 3e,g; *p* < 0.01) yet promoted these molecules in the slow muscle (Figure 3f,h; *p* < 0.05). 

### 3.3. SC Differentiation in Response to Lys Deficiency

**Figure 4:**
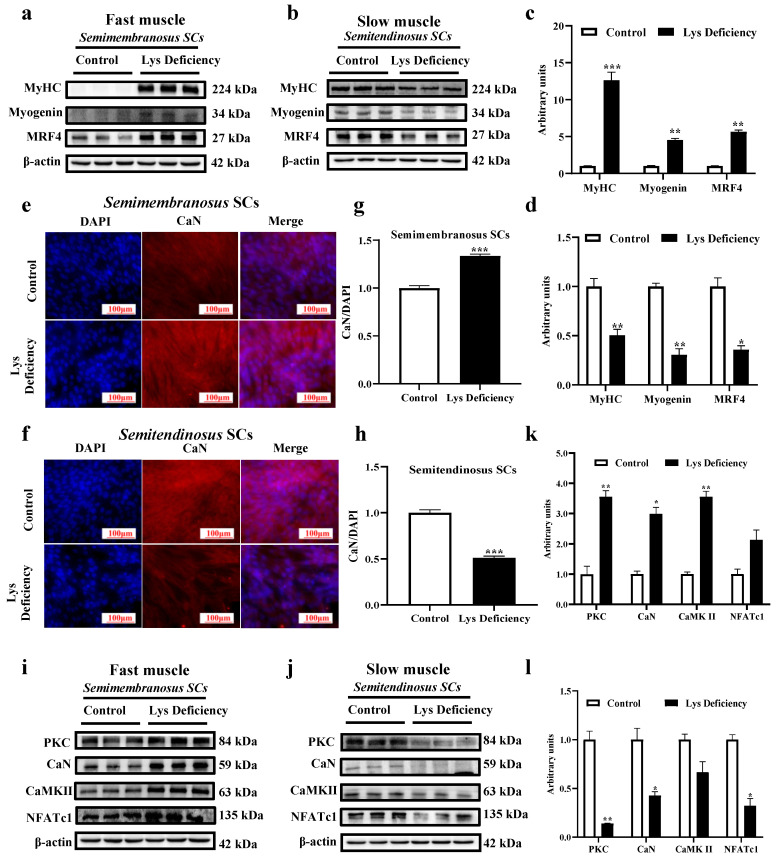
Effects of Lys deficiency on the differentiation and Wnt/Ca^2+^ pathway in *semimembranosus* and *semitendinosus* SCs. Expression of MyHC and differentiation marker proteins (myogenin and MRF4) in porcine fast (*semimembranosus*; **a**,**c**) and slow (*semitendinosus*; **b**,**d**) muscle SCs after 48 h Lys deficiency. CaN fluorescence intensity in 48 h Lys deficiency-treated porcine fast (*semimembranosus*; **e**,**g**) and slow (*semitendinosus*; **f**,**h**) muscles. Expression assay of Wnt/Ca^2+^ pathway key proteins (PKC, CaN, CaMKII, and NFATc1) in fast (*semimembranosus*; **i**,**k**) and slow (*semitendinosus*; **j**,**l**) muscle SCs after 48 h Lys deficiency. * indicates *p* < 0.05, ** indicates *p* < 0.01, *** indicates *p* < 0.001.

SCs isolated from both fast and slow muscles were studied to determine the myogenic differentiation mechanism in response to Lys deficiency. Consistently, Lys deficiency for 48 h mediated the MRFs in an opposite fashion between fast and slow muscle-derived SCs. It promoted the differentiation of modulating molecules in fast muscle SCs (Figure 4a,c; *p* < 0.05) and suppressed those expressions in slow muscle SCs (Figure 4b,d; *p* < 0.05). The immunofluorescence intensity of CaN/DAPI was upregulated in Lys-deficient cultured *semimembranosus* SCs (Figure 4e,g) but was significantly reduced in *semitendinosus* SCs (Figure 4f,h). Besides CaN, the protein expressions of other key molecules including PKC, CaMKII, and NFATc1 were also stimulated in *semimembranosus* SCs when cultured in the Lys-deficient medium (Figure 4i,k). However, the expressions of these molecules were all suppressed in expression in *semitendinosus* SCs under the same Lys-deficient condition (Figure 4j,l).

**Figure 5:**
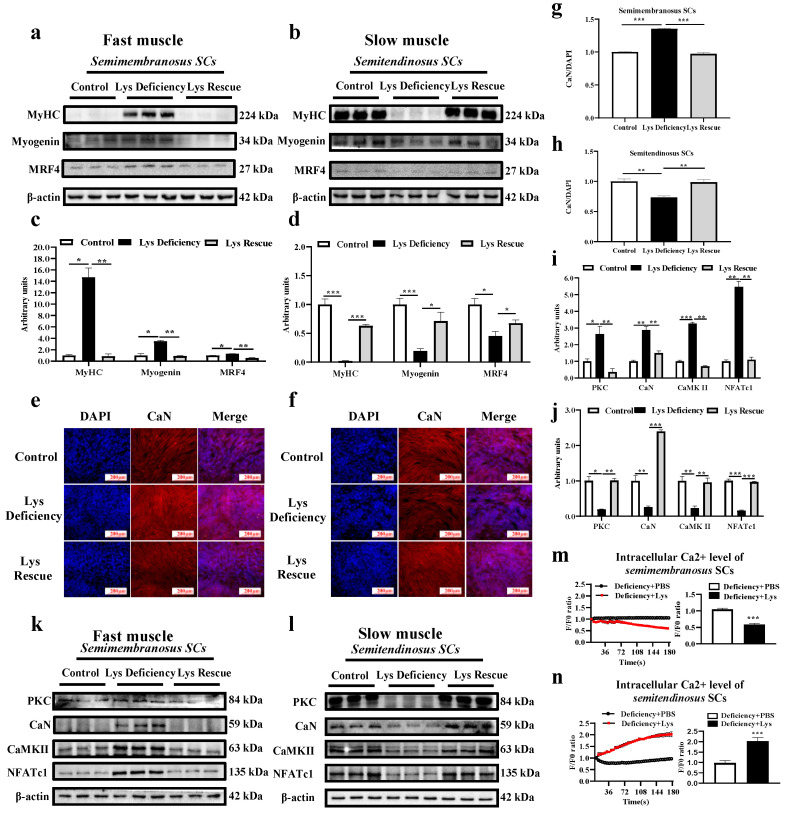
Effects of Lys on the differentiation, calcium concentration, and Wnt/Ca^2+^ pathway in *semimembranosus* and *semitendinosus* SCs. Expressions of MyHC and key proteins (myogenin and MRF4) of the myogenic factor family in fast (*semimembranosus*; **a**,**c**) and slow (*semitendinosus*; **b**,**d**) muscle SCs detected upon study completion (96 h). CaN fluorescence intensity in fast (*semimembranosus*; **e**,**g**) and slow (*semitendinosus*; **f**,**h**) muscle SCs. Wnt/Ca^2+^ pathway key proteins (PKC, CaN, CaMKII, and NFATc1) expression in fast (*semimembranosus*; **i**,**k**) and slow (*semitendinosus*; **j**,**l**) muscle SCs. Intracellular Ca^2+^ concentration changes within and at 120 s upon Lys restoration in Lys-deficient fast (*semimembranosus*; **m**) and slow (*semitendinosus*; **n**) muscle SCs. * indicates *p* < 0.05, ** indicates *p* < 0.01, *** indicates *p* < 0.001.

In agreement with the in vivo model, Lys rescue induced a significant reduction in MyHC and MRFs proteins in *semimembranosus* SCs at 96 h compared with the Lys-deficient status. The opposite pattern was observed in *semitendinosus* SCs (Figure 5a–d). These results showed that Lys deficiency promoted myogenic differentiation in *semimembranosus* SCs, which could be repressed by Lys rescue. The adverse mechanism was observed in the *semitendinosus* SCs. CaN, together with other key molecules (PKC, CaN, CaMKII, and NFATc1) involved in controlling the Wnt/Ca^2+^ pathway, was reduced by Lys deficiency in the slow muscle at 96 h and got promoted when sufficient Lys was supplemented (Figure 5f,h,l,j; *p* < 0.05). The fast muscle displayed an opposite modulation in the Wnt/Ca^2+^ pathway upon Lys deficiency and rescue treatment (Figure 5e,g,k,i; *p* < 0.05). Muscle-type-dependent modulations of intracellular Ca^2+^ were discovered in in vitro SC models. Lys rescue remarkably decreased intracellular Ca^2+^ in *semimembranosus* SCs but increased Ca^2+^ in the *semitendinosus* SCs (Figure 5m,n).

## 4. Discussion

The amino acids pool constitutes a major reservoir of nitrogen sources for supporting skeletal muscle growth. Understanding the modulating mechanism of myogenic differentiation and growth regarding Lys deficiency holds promise in guiding a better myogenic differentiation potential in skeletal muscles. Lys, one of the essential amino acids for swine, could exert an important signaling pathway in mediating SC differentiation to facilitate the progression into myofibers [15,20,21]. However, there is a paucity of knowledge concerning how *semimembranosus* and *semitendinosus* developments are affected by dietary Lys. To fill this gap, we employed both in vivo and in vitro models to evaluate the differentiation responses to Lys deficiency in two different muscle types. 

### 4.1. Lys Affects Fast and Slow Muscle Development

In our study, dietary Lys deficiency greatly compromised the growth of both *semimembranosus* and *semitendinosus* muscles in young piglets. However, this could be reversed by Lys supplementation, which demonstrated the indispensable role of Lys in early development. This phenomenon agreed with previous investigations that determined insufficient Lys caused nitrogen dysbiosis and skeletal muscle development disorder. Re-supplementing sufficient Lys in the diet counteracted the detrimental effects and restored healthy growth [21,22,23]. However, in fattening pigs, when the dietary Lys level was reduced, *semimembranosus* quality traits regarding weight, appearance, and pH remained unaffected [24]. It is noteworthy that the growth restriction induced by Lys deficiency was more significant in fast muscles than in slow muscles in our study. Verdijk reported that the fast muscle is more hypersensitive to nutritional alterations, which could be the reason that it was hindered more than the slow muscle during development [25]. 

### 4.2. Expressions of Myogenic Modulating Factors and MyHC in Fast and Slow Muscle SCs

SCs are embedded between the sarcolemma and the basement membrane in direct contact with myofiber. This special location provided an acceptable microenvironment to facilitate the myogenesis process [26]. In the current study, fast muscle-derived SC accelerated myofiber differentiation when cultured with a Lys-deficient medium, while the slow muscle SCs executed a decelerated differentiation. This mechanism was further verified by Lys re-supplementation: fast muscle SCs slowed down their differentiation after being transferred to a sufficient Lys medium, while slow muscle SCs accelerated their differentiation. The current results compensated Khodabukus’s finding that the differentiation properties of SCs follow the parental origin [6]. 

In general, myogenic modulating factors present a series of fate-determining transcriptional factors in the entire cell lineage and the key proteins of MRFs, targeting different points to regulate routine muscle renewal from proliferation, differentiation, and phenotype formation. Interestingly, the MRFs’ proteins also express different patterns in fast and slow muscles resembling responses in SCs to Lys deficiency, and the opposite activities of MRFs are in line with previous studies [9,27]. Notably, unlike slow muscles, MyHC and MRFs were not sensitive to dietary Lys changes in fast muscles on day 29; this could be due to the dilution from other muscular processes that are regulated by MRFs. Different sensitivities of fast and slow muscles to external stimulus were reported previously, showing that slow electrical stimulation on fast muscles significantly upregulated myogenin, but fast muscle-type electrical stimulation did not induce any changes in slow muscles [28]. In another study, denervated muscle extract treatment in mice activated myofiber hypertrophy and increased myogenic activity in *semitendinosus* muscles but caused type II muscle atrophy in *semimembranosus* muscles [29]. 

### 4.3. Wnt/Ca^2+^ Pathway and Myogenic Differentiation

Wnt-involved signaling cascades were shown to exert important roles through all developmental stages in muscles, including satellite cell proliferation, niche renewal, the initiation of differentiation by MRF modulation, and other broad physiological processes [30,31,32]. In SCs, The Wnt/Ca^2+^ pathway is known as an important driver for differentiation and muscular cell migration by regulating cell polarity. Here, we isolated both SCs from fast and slow muscles to compare how this signaling reacts to Lys deficiency. As expected, Lys deficiency positively modulated both Ca^2+^ concentrations and key molecules in the Wnt/Ca^2+^ pathway (PKC, CaN, CaMKII, and NFATc1) in fast muscle SCs but reduced both parameters in its slow counterpart. Regulations on the Wnt/Ca^2+^ pathway were sensitive to the re-supplementation of sufficient Lys with a repressing effect in the fast muscle SCs and a boosting effect in their slow counterpart. These results supported our previous statement that the Wnt/Ca^2+^ pathway is greatly associated with muscle differentiation and exhibits fiber-type-dependent responses. Interestingly, even comprising most mature muscle fibers, the further detection of Ca^2+^ concentration and Wnt/Ca^2+^ pathways in slow and fast muscles in pigs corroborated the changes discovered in the SCs. It is worth noting that mature myofibers possess similar mechanisms of Ca^2+^ regulation with their local SCs, and the Wnt/Ca^2+^ pathways leading to the dephosphorylation of NFAT and subsequent nuclear entry modulate not only myogenesis but also oxidative metabolism, myoblast fusion, and muscle regeneration [33].

Despite the reciprocal inhibition effect between Wnt/Ca^2+^ and Wnt/β-catenin pathways, both can modulate SC myogenic differentiation [22,34]. Liu et al. reported that osteogenic differentiation can be promoted through the activation of the non-canonical Wnt5a/Ca^2+^ pathway by β-catenin suppression [35]. However, the role of the Wnt/β-catenin pathway in SC myogenic differentiation remains debatable. Otto et al. stated that the activation of the Wnt/β-catenin pathway can promote SC proliferation while repressing their differentiation [12]. However, other research groups highlighted that the excessive activation of this pathway could advance the differentiation process which further impairs the SCs pool and induces myogenic disorder [36]. Hence, as an antagonistic pathway against Wnt/β-catenin, the modulating role of Wnt/Ca^2+^ on myogenic differentiation is still unknown. As suggested by previous research, the blockage of the Wnt5a/Ca^2+^ pathway led to an impeded growth of tongue muscle [37]. Furthermore, Wnt5a, when regulated by muscle anabolic regulator 1 (MAR1), elevated the muscular cross-sectional area and strength in aged mice [14]. In other studies, CaN and CaMK stimulated the downstream target genes to promote slow muscle myogenesis [38,39], while Wnt5a was proved to promote the slow muscle as a stimulator in chicken SCs in an in vitro model [40]. All the above studies revealed that the Wnt/Ca^2+^ pathway participates in the mediation of skeletal myogenic differentiation and potentially initiates a muscle-type-dependent effect.

Skeletal muscles are important organs involved in metabolic regulation and body movement. Maintaining the dynamic balance of the body at different stages of the body is a prerequisite for normal skeletal muscle development. Any disruption to this balance could result in the retardation of muscle growth [41]. This statement was verified in the current study where Lys deficiency remarkably hindered SC myogenic differentiation by a positive or negative muscle-type-dependent modulation of the Wnt/Ca^2+^ pathway. Peng et al. discovered that the stimulation of the Wnt/Ca^2+^ pathway not only promoted SC differentiation but also suppressed SC proliferation, resembling the mechanism of the Wnt/β-catenin pathway. The excessive activation of the Wnt/Ca^2+^ pathway impaired the SCs pool to further slow down skeletal muscle growth. Hence, the Wnt/Ca^2+^ pathway plays a key role in balancing myogenic differentiation. In addition, the cellular Ca^2+^ concentration varies in fast and slow muscles due to the different contraction and metabolic features [42]. This was validated in the current study where Lys deficiency disrupted the Ca^2+^ equilibrium in fast and slow muscle SCs, leading to further disruption in their differentiation through the Wnt/Ca^2+^ pathway. Also, Lys deficiency significantly increased the fast muscle SC differentiation at 96 h but did not affect the overall muscular differentiation in the pig study. Hence, it is speculated that long-term Lys deficiency greatly reduced the population of SCs in the fast muscle, which weakened the overall degree of differentiation. 

## 5. Conclusions

**Figure 6:**
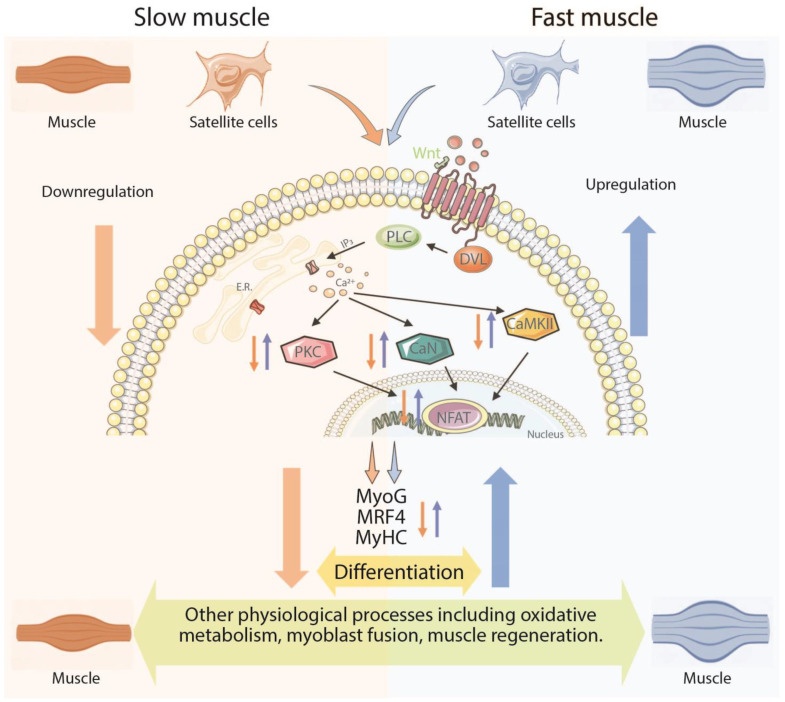
The current study depicts different Lys-manipulated mechanisms on the Wnt/Ca^2+^ pathway and myogenic differentiation in fast (*semimembranosus*) and slow (*semitendinosus*) muscle and satellites cells. Lys deficiency stimulates the Wnt/Ca^2+^ pathway and myogenic differentiation in fast muscle, while it suppresses these biological processes in slow muscle. (SC: satellite cells; MyoG: myogenin; MRF4: myogenic regulatory factor 4; PKC: protein kinase C, CaN: calcineurin; CaMKII: calcium/calmodulin-dependent protein kinase II; NFAT: nuclear factor of activated T; PLC: phospholipase C; DVL: disheveled).

The current study disclosed the different mechanisms of myogenic differentiation in response to dietary Lys levels. In the slow muscle SCs, Lys deficiency hindered differentiation and muscle development by suppressing the SCs Wnt/Ca^2+^ pathway. In fast muscle SCs, the development was repressed by Lys deficiency but through an opposite mechanism of the SCs Wnt/Ca^2+^ pathway, and myogenic differentiation was overstimulated and to a greater degree than their slow counterpart. When sufficient Lys was achieved, the fast muscle SCs-derived Wnt/Ca^2+^ pathway was downregulated, and the overdifferentiation terminated. Furthermore, the Wnt/Ca^2+^ pathway in the slow muscle SCs was stimulated to further the myogenic differentiation process, and the repression of skeletal muscle growth was diminished (Figure 6). In summary, our findings revealed the different mechanisms of myogenic differentiation in response to dietary lysine levels in fast and slow muscles.

## Data Availability

Data will be made available on request.

## Supplementary Materials

The following supporting information can be downloaded at: https://www.mdpi.com/article/10.3390/cells13070650/s1, Ingredient composition and nutritional levels of the diets (air-dry basis; Table S1); Primary and secondary antibodies used in the Western blotting (Table S2); Primary and secondary antibodies used in the immunofluorescence staining (Table S3).

## Abbreviations

SCs, satellite cells; MyHC, myosin heavy chain; MRFs, myogenic regulatory factors; MyoD, myoblast determination protein; MyoG, myogenin; MRF4, myogenic regulatory factor 4; PCP, planar cell polarity; Lys, lysine; PMSF, phenylmethanesulfonyl fluoride; SDS-PAGE, sodium dodecyl-sulfate polyacrylamide gel electrophoresis; PVDF, polyvinylidene fluoride membranes; DAPI, 4′,6-di- amidino-2-phenylindole; MAR1, muscle anabolic regulator 1; PKC, protein kinase C; CaN, calcineurin; CaMKII, calcium/calmodulin-dependent protein kinase II; NFAT, nuclear factor of activated T; PLC, phospholipase C; DVL, disheveled.
