# Supplementary material for: Lysine Distinctively Manipulates Myogenic Regulatory Factors and Wnt/Ca2+ Pathway in Slow and Fast Muscles, and Their Satellite Cells of Postnatal Piglets
Source: Cells. 2024 Apr 8;13(7):650. doi: 10.3390/cells13070650 (PMC11011516; doi:10.3390/cells13070650)
Supplement: Supplementary file 1 [file cells-13-00650-s001.zip › cells-2783165-supplementary.pdf]

Table S1 Ingredient composition and nutritional levels of the diets (air-dry basis)

Item	Content, %	
	Control	Lys Deficiency
Ingredients		
Corn	33.66	33.90
Soybean meal	24.00	24.00
Extruded corn	20.00	20.00
Flour	10.00	10.00
Fermented soybean meal	4.00	4.00
Soybean oil	2.00	2.00
Dicalcium phosphate	0.80	0.80
Limestone power	0.72	0.85
Mildew preventive	0.05	0.05
Sodium chloride	0.40	0.40
**L-Lysine hydrochloride**	**0.37**	**0.00**
Premix[1]	4.00	4.00
Total	100.00	100.00
Nutritional levels		
Digestible energy, MJ/kg	14.23	14.23
Crude protein[2]	19.15	19.13
**Lysine**	**1.30**	**0.86**
Methionine + cystine	0.58	0.58
Threonine	0.71	0.71
Tryptophan	0.23	0.23
Calcium	0.53	0.53
Phosphorus	0.47	0.47

[1] The premix provided the following nutrients per kilogram of diet: Fe 101.00 mg; Cu 15.00 mg; Mn 42.00 mg; Zn 102.00 mg; I 0.60 mg; Se 0.30 mg; VA 10500.00 IU; VD_3 2100.00 IU; VK_3 3.50 mg; VB_1 2.24 mg; VB_2 9.80 mg; VB_6 2.80 mg; VB_{12} 0.05 mg; Folic acid 1.04 mg; Niacin 35.00 mg.

[2] Crude protein was analyzed. Other values were calculated.

Table S2. Primary and secondary antibodies used in the Western blotting

**Antibody/type**	**Source**	**Manufacturer**	**Catalogue number**
CaMKII/primary	Rabbit	Sangon	D120336
CaN/primary	Rabbit	Sangon	D122013
PKC/primary	Rabbit	Sangon	D121794
NFATc1/primary	Mouse	Santa Cruz Biotechnology	sc-7294
MyoD/primary	Rabbit	Santa Cruz Biotechnology	sc-304
Myogenin/primary	Rabbit	Santa Cruz Biotechnology	sc-12732
MRF4/primary	Mouse	Santa Cruz Biotechnology	sc-514379
MyHC/primary	Rabbit	Santa Cruz Biotechnology	sc-376157
β-actin/primary	Mouse	Santa Cruz Biotechnology	sc-8432
Goat Anti-Rabbit IgG/secondary	-	Earthox LLC	#E030120
Goat Anti-Mouse IgG/secondary	-	Earthox LLC	#E030110

Table S3. Primary and secondary antibodies used in the Immunofluorescence staining.

**Antibody**	**Source**	**Manufacturer**	**Catalogue number**
MyoD/primary	Rabbit	Santa Cruz Biotechnology	sc-304
CaN/primary	Rabbit	Sangon	D122013
Goat Anti-Mouse IgG/secondary	-	Jackson	115-545-003
Goat Anti-Rabbit IgG/secondary	-	Jackson	111-165-045
